# No evidence of differential impact of sunflower and rapeseed oil on biomarkers of coronary artery disease or chronic kidney disease in healthy adults with overweight and obesity: result from a randomised control trial

**DOI:** 10.1007/s00394-022-02810-5

**Published:** 2022-04-05

**Authors:** Katie Nicol, Bahareh Mansoorian, Agnieszka Latosinska, Aimilia Koutroulaki, Bill Mullen, Emilie Combet

**Affiliations:** 1 Human Nutrition, School of Medicine Dentistry and Nursing, College of Medical, Veterinary and Life Sciences, University of Glasgow, New Lister Building, Glasgow Royal Infirmary, Alexandra Parade, Glasgow, G31 2ER UK; 2 Institute of Cardiovascular and Medical Sciences, College of Medical, Veterinary and Life Sciences, University of Glasgow, Glasgow, UK; 3 Mosaiques Diagnostics GmbH, Hannover, Germany

**Keywords:** Monounsaturated fatty acids, Seed oils, Dietary fat, Cardiovascular, Cardiometabolic, Biomarkers, Proteomic, Rapeseed, Sunflower

## Abstract

**Purpose:**

The perceived benefits and risks associated with seed oil intake remain controversial, with a limited number of studies investigating the impact of intake on a range of compounds used as cardiometabolic markers. This study aimed to explore the proteomic and cardiometabolic effects of commonly consumed seed oils in the UK, with different fatty acid profiles.

**Methods:**

In a parallel randomised control design, healthy adults (*n* = 84), aged 25–72 with overweight or obesity were randomised to one of three groups: control (habitual diet, CON); 20 mL rapeseed oil per day (RO), or 20 mL sunflower oil per day (SO). Blood, spot urine and anthropometric measures were obtained at 0, 6 and 12 weeks. Proteomic biomarkers analysis was conducted for coronary arterial disease (CAD) and chronic kidney disease (CKD) using capillary electrophoresis coupled to mass spectrometry (CE-MS). Blood lipids, fasting blood glucose, glycative/oxidative stress and inflammatory markers were also analysed.

**Results:**

No differences in change between time points were observed between groups for CAD or CKD peptide fingerprint scores. No change was detected within groups for CAD or CKD scores. No detectable differences were observed between groups at week 6 or 12 for the secondary outcomes, except median 8-isoprostane, ~ 50% higher in the SO group after 12-weeks compared to RO and CON groups (*p* = 0.03).

**Conclusion:**

The replacement of habitual fat with either RO or SO for 12 weeks does not lead to an improvement or worsening in cardiovascular health markers in people with overweight or obesity.

**Trial registration:**

Trial registration clinicaltrials.gov NCT04867629, retrospectively registered 30/04/2021.

**Supplementary Information:**

The online version contains supplementary material available at 10.1007/s00394-022-02810-5.

## Introduction

The health benefits of olive oil consumption on cardiovascular disease risk have been recognised by the Food and Drug Administration (FDA) and European Food Safety Authority (EFSA) [1]; and attributed to the high amounts of mono-unsaturated fatty acids (MUFA) (chiefly oleic acid), and phenolic compounds [2, 3]. There is supportive evidence for 20 g of an oil containing high levels of oleic acid, when replaced for fats and oils higher in saturated fats (SFA) to reduce the risk of coronary heart disease (CHD) [4]. The replacement of SFA with polyunsaturated fatty acids (PUFA) was also shown to reduce CHD events; with a 13% lower risk of CHD for each 5%E (% energy intake) greater PUFA intake in place of SFA [5].

Plant oils intake (in place of sources of SFA) exerts cardioprotective effects by decreasing blood lipids, recognized as traditional CVD risk factors. There is limited evidence supporting the greater impact of any specific plant oil on blood lipids, with a network meta-analysis presenting the superiority of unsaturated fatty-rich oils over sources of saturated fats in the modulation of blood lipids, with the differential effect of each oil type for the different blood lipid marker [6]. A 10% isocaloric substitution of palm oil with rapeseed oil (RO) and sunflower oil (SO) reduced total cholesterol (TC) by 0.2 and 0.1 mmol/L respectively, corroborating guidelines for replacement of SFAs with unsaturated fatty acids, based on their impact on blood markers [6].

Primary outcome markers currently used to evaluate the impact of diet components, such as seed oils, on health (TC, HDL cholesterol, LDL cholesterol, or oxidized LDL) are based on association with mortality and do not necessarily reflect effects on the early stage of the disease itself [7]. As such, this is a limitation of research aiming toward genuine prevention. Urinary proteomics is a valuable tool to measure changes as a direct result of disease progression or treatment, accounting for pathophysiologic changes. Urinary proteomics has been successfully piloted in the diagnosis of renal disease, transplant rejection, and cancer [8]. We have carried out several studies into the identification of biomarkers of a range of these diseases. They include biomarkers of chronic kidney disease (CKD) and coronary artery disease (CAD) [9–12].

Vegetable/seed oils are recommended to replace other dietary fats rich in SFA based on their fatty acids composition (PUFA/MUFA rich), which has demonstrated a favourable impact on coronary heart events [5]. The role of phenolic compounds in the disease risk reduction associated with olive oil consumption is less clear. In a recent randomised controlled trial [13], intake of olive oil (20 mL per day over 6 weeks) lowered urinary proteomic biomarkers of CAD, but not CKD, regardless of phenolic content. This reinforced the proposition that the fatty acid profile of the oil is a driving factor for its health-promoting activity and disease prevention.

Olive oil is the main source of added fat in the Mediterranean diet, and a key component distinguishing the Mediterranean diet from other dietary patterns [14]. However, it is not commonly consumed as part of a UK diet, with food balance sheets indicating a 2.9 g of fat/capita/day from olive oil, versus 6.7 g/capita/day from sunflower oil and 24.1 g/capita/day from rapeseed and mustard oil [15]. The relative high cost of imported olive oil compared to locally grown seed oils is one barrier to its wider adoption in the UK. Unlike RO and SO, olive trees only grow in a Mediterranean like climate and therefore must be imported to countries out with that area [16]. Both age and income level are key factors that influence the frequency and amount of olive oil consumed in the UK, with higher income brackets and lower age groups (below 50 years old) contributing most to frequent and occasional olive oil consumption in the UK [17].

The fatty acid profile of vegetable oils varies considerably, with RO having the highest MUFA content among seed oils (59 g/100 g RO, compared to 20.5 g/100 g SO), and SO being the richest in n-6 PUFA (63 g/100 g SO, almost exclusively Linoleic Acid (LA), compared to 20 g/100 g RO) [18]. Based on the fatty acid profile, MUFA-high RO could be an alternative to olive oil (73 g MUFA/100 g oil) [2].

Despite the number of intervention studies focussing on cardiometabolic effects of seed oils (measuring mostly proxy markers) [19–22], no clear conclusion can be drawn regarding which is more beneficial on cardiovascular health. With intervention studies focussing on groups differing in terms of gender, age, body composition, dietary habits, as well as oil doses and duration, it is difficult to clearly recommend a particular dietary oil regimen to any given group. The role of different fatty acid profiles on cardiovascular health markers needs to be elucidated, not only to inform guidelines but also to contribute to the production of foods with optimal fatty acid profiles. The observations of Silva et al. [13] and the current ambiguous findings on the cardiometabolic impact of seed oils, emphasise the need to integrate the state of the art, highly specific proteomic biomarker for disease analysis with nutrition research.

The primary aim of this study was to evaluate the impact of a 12-week intervention with rapeseed oil and sunflower oil, two commonly consumed seed oils with divergent fatty acid profiles, on urinary proteomic biomarkers specific for CAD and CKD. Secondary outcomes measured included plasma lipids profile, fructosamine, and inflammatory markers.

## Methods

### Recruitment

Participants (*n* = 84) aged 25–75 years were recruited from the community in the Greater Glasgow area (Scotland, UK) between July and November 2015, using advertising via the British Broadcasting Corporation (BBC), poster and social media calls for participants. Participants were self-reported healthy adults, with no current diagnosed illness, not pregnant or lactating, and not allergic to any vegetable oils and vegetable oil-derived products. A criterion for eligibility was a BMI over 25 kg/m^2^ and/or a waist circumference > 88 cm for women and > 102 cm for men. Other exclusion criteria included a history of chronic disease of the gastrointestinal tract and taking any form of medication other than the contraceptive pill. Smokers were not excluded from the study.

### Study design

The intervention followed a parallel randomised control design. The intervention duration was 12 weeks, with a mid-point assessment at 6 weeks. Randomisation to groups; rapeseed / canola oil (RO), sunflower oil (SO) or habitual diet (CON) was performed using a block stratified allocation based on age (under or over 45) and BMI (under or over 30 kg/m^2^) and was carried out remotely via phone using a pre-set list with block sizes of 6 allocations.

Our previous study with olive oil in healthy volunteers [13] showed a within-group change ΔCAD = 0.38 (SD 0.26) in participants with a large waist. Based on the assumption that control participants would show no changes in CAD score (ΔCAD = 0.00), 8 participants per group would be required to detect a similar change (α = 0.05, β = 80%, F test/ANOVA) or a total sample size of *n* = 24, and *n* = 28 would be required to detect a smaller (half) effect (ΔCAD = − 0.19) or a total sample size of *n* = 84. Allowing for a 15% dropout, sample size was set at *n* = 100 participants.

The study was approved by the Ethics Committee of the College of Medical, Veterinary & Life Sciences, University of Glasgow (200140146) and retrospectively registered on clinicaltrials.gov NCT04867629 (30th April 2021). Protocols were according to the Declaration of Helsinki, and all participants provided informed consent at recruitment.

### Intervention

Participants allocated to either RO or SO groups were instructed to consume 20 mL of the oils, uncooked, as a partial replacement to their habitual fat intake. Participants were provided with suggestions on incorporating the oils into their meals, including but not limited to as dressing, on bread, in sandwiches, or in food (e.g. pasta/rice) after cooking. Dietary oils provided for the study were both commercially available oils. The rapeseed oil (Hillfarms, Suffolk–extra virgin cold-pressed) contained 55 g MUFA and 24 g PUFA and 6.5 g SFA per 100 ml, and sunflower oil (Sainsbury's own) contained 25.8 g MUFA and 56 g PUFA and 10.1 g SFA per 100 ml. Participants in the control group were asked to not change any aspect of their habitual diet.

The oil supply (labelled A or B) was provided for 6 weeks in three amber glass bottles containing 300 mL each. At week 6 and 12, participants visited the department to return the oil bottles to be weighed for compliance and for a follow-up to ensure they adhered to the test diet and kept a stable body weight during intervention periods. Adherence was assessed via scrutiny of intake logs and weighing the amount of unconsumed oil returned at the 6-week and 12-week visits.

### Dietary assessment

Participants were asked to record prospectively their entire dietary intake for 2 consecutive days before their baseline visit. This included estimated portion sizes, time of consumption and cooking methods for each meal of the day and any snacks. Food portions were estimated using standard units and household measures. Participants were encouraged to include brand names of any food products or pre-packaged foods. Participants were asked to replicate the same 2-day intake before their follow-up visits in week 6 and week 12. Food diaries were analysed using Nutritics Nutrition Analysis Software (Nutritics, 2019) and then exported to Microsoft Excel (Version 365) for further analysis. Food diaries were incomplete for 2 participants and excluded from the analysis (SO = 1, CON = 1).

All participants were asked to complete the EPIC-Norfolk Food Frequency Questionnaire (FFQ) during their baseline visit. The questionnaire consists of a 130-item food list. Participants were requested to select an appropriate frequency of consumption for each line from the nine frequency categories. The questionnaire lines were either individual foods, combinations of individual foods or food types. The FFQ food list is based on items commonly consumed in the UK. The FFQ was designed to estimate habitual intake over the previous year and nutrients were computed using the FETA software. Supplement use was not included in the calculation of nutrient intakes. There were 7 participants with FFQ missing or incomplete at baseline (RO = 1, SO = 2, CON = 4).

### Sample collection

Spot urine samples were collected from all participants at all time points for measurement of the primary outcome (proteomic biomarker score). Urine was collected in a plastic tube, mid-flow, as the second urination of the day at baseline, 6 and 12 weeks. Samples were split into aliquots and frozen at − 80 °C until analysis.

Fasting venous blood was collected for the assessment of secondary outcomes, at baseline, 6 weeks and 12 weeks. Blood samples could not be obtained from 8 participants at baseline (3 RO, 3 SO, 2 CON), 11 participants at week 6 (5 RO, 3 SO, 3 CON) and 9 participants at week 12 (4 RO, 3 SO, 2 CON). Blood samples were centrifuged at 2140×*g* for 5 min at 4 °C, and the plasma was split into aliquots and stored at − 80 °C until analysis.

Analysis of blood and urine samples were carried out blinded.

### Anthropometric measures

Fasted body weight was measured to the nearest 0.1 kg using an electric scale (Tanita Body Composition Analyzer TBF-310), height was measured to the nearest millimetre using a stadiometer (Tanita Leicester Height Measure), and waist circumference was measured using a non-elasticated tape at the smallest abdominal position between the lowest rib and iliac crest (with the participant standing after an expiration). Blood pressure was measured using a digital automatic blood pressure monitor (Omron M5-1) with the participant seated after 30 min of rest. Valid blood pressure data is missing for 9 participants at baseline (3 RO, 2 SO, 4 CON), 8 at week 6 (2 RO, 2 SO, 4 CON) and 3 (2 RO, 1 CON) at week 12. All measurements were done in duplicate.

### Proteomic analyses by CE-MS

CE-MS has been successfully used in the analysis of naturally occurring peptides in urine. A 700µL aliquot of urine was thawed immediately before use and diluted with 700µL urea buffer [2 M urea (Sigma Aldrich), 0.1 M NaCl (Fisher Scientific), 10 mM NH_4_OH (Fisher Scientific), and 0.02% sodium docecyl sulfate (Sigma Aldrich), pH 10.5]. Prior to CE-MS analysis, the samples were filtered using Centrisart filters (20 kDa molecular weight cut-off; Sartorius, Göttingen, Germany) by centrifugation (IEC GP8) at 2266xg for 60 min at 4 °C to remove higher molecular weight proteins, followed by the desalting step using a PD-10 column and ammonia buffer (GE Healthcare, Germany). Subsequently, samples were lyophilized and resuspended in 10 µl HPLC-grade water before CE-MS analysis, as previously described [13, 23]. CE-MS analysis was performed with a P/ACE MDQ capillary electrophoresis system (Beckman Coulter, USA) coupled to a micro-TOF–MS (Bruker Daltonic, Germany). The electrospray ionization interface from Agilent Technologies (Palo Alto, CA, USA) was applied. Spectra are accumulated every 3 s, over a range of m/z 350–3000 [24]. Mass spectral ion peaks representing identical molecules at different charge states are deconvoluted into single masses using MosaiquesVisu software [25]. Normalization of the CE-MS data was based on 29 collagen fragments that are not affected by disease and serve as internal standards [11, 26]. The final result is a peak list characterising each peptide by its mass [kDa] and normalised CE migration time [27, 28]. After normalisation, all detected peptides were transferred to a Microsoft SQL database used for further analysis and comparison of individual samples. Biomarkers are generated from case versus control studies where the compiled data from the two groups were compared [9–12]. Peptides that are significantly different between groups are combined into a classifier for the selected disease. In the case of CAD, this is made up of 238 peptides [10]. These peptides are combined to produce a score for each sample. From the case versus control data a “score” above a set value is deemed to be positive for the disease and negative below this value. In the case of CAD, the threshold score is − 0.140 [10], for CKD, it is 0.343 [29].

### Plasma biomarkers

Plasma TC, TAG and HDL-C were all measured using CHOD-PAP method, GPO-PAP method and direct clearance method assays modified for a 96-well plate using plasma lipid kits originally designed for an RX MONZA (RANDOX, Crumlin, UK). LDL-C was determined with the Friedewald equation [LDL = Cholesterol − HDL − (Triglycerides/2.2)] [30]. Randox Glucose GOD-PAP kit was used with modification for a 96-well plate to determine fasted blood glucose concentrations (RANDOX, Crumlin, UK).

The early glycation marker fructosamine and receptors for advanced glycation end-products (RAGE) were measured with the Nitroblue tetrazolium (NBT) assay and a Human RAGE ELISA (RayBioTech, Georgia, USA) respectively. To measure the fluorescence intensity of the advanced glycation end-products, serum samples were diluted 1:5 with phosphate-buffered saline (PBS). To detect the signal, the spectrofluorometric detector was set to emission 440 nm and excitation 370 nm (SpectraMac M2e with SoftMax Pro software). Plasma matrix metalloproteinase-9 (MMP-9) levels were measured with the Human MMP-9/TIMP-1 Complex DuoSet ELISA (R&D Systems Inc, Abingdon, UK). TNF-α and IL-6 were analysed using commercially available ELISA kits (ThermoFisher Scientific Inc). All CV% were < 20%.

### Urine biomarkers

Spot urine samples were analysed for 8-isoprostane using a commercial ELISA kit (Oxford Biomed). The results were then normalised against urinary creatinine levels using the Jaffe method colorimetric assay.

### Statistical analysis

Statistical analyses were conducted using SPSS version 24 (IMB) and RStudio packages ggplot2 and ggally (RStudio). Normality was assessed using the Shapiro–Wilk *W* test, and data are expressed as mean (SD) or as median (IQR) accordingly. Between-group baseline differences in variables and changes from baseline at week 6 and 12 were compared with one-way ANOVA or Kruskal–Wallis tests depending on normality. One-way ANCOVA was performed to compare differences in cardiometabolic biomarkers between RO, SO and CON at week 6 and 12 with covariate adjustment for baseline levels. Within-group differences were assessed by one-way repeated measures ANOVA or Friedman test based on normality. If sphericity was not assumed, the Greenhouse–Geisser *p* value was used. Alpha was set at 0.05.

## Results

**Fig. 1:**
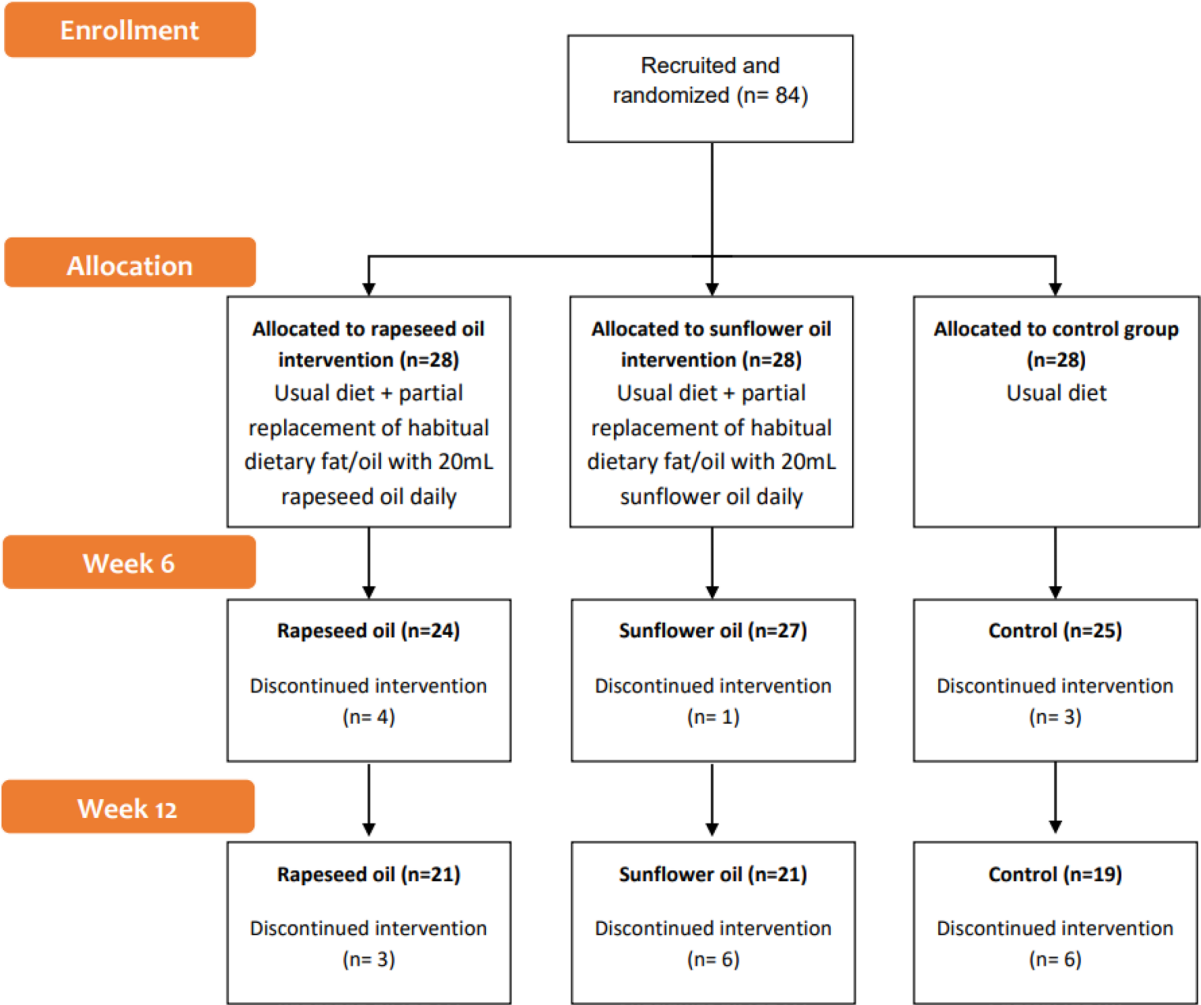
Flowchart of participants from baseline (week 0) until the end of intervention (week 12)

Eighty-four participants were recruited and randomised (*n* = 28 per group, Fig. 1). Out of those, *n* = 61 completed the study, with a drop-out rate of 8% at 6 weeks and 27% at 12 weeks. Of those who completed at least 6 weeks of the intervention (*n* = 76, 34 men, 42 women), median age was 43 (IQR 32–54) with a median BMI of 29 (IQR 27–31). Both men (71%) and women (67%) who were randomised had a large waist circumference at baseline, above the level 2 risk cut-offs of 88 cm for women, and 102 cm for men (men, *n* = 24/34, median 108 cm, IQR 105–114; women, *n* = 28/42, median 102 cm, IQR 96–106). Prevalence of overweight was 49% and obesity was 40%. There were no differences in body composition (as kg of body mass or as unit of BMI) between groups at baseline (Table 1). Group median systolic (129 mmHg, 121–143) and diastolic (81 mmHg, 76–89 IQR) blood pressure was higher than optimal levels at baseline with 14 participants falling above the cut-offs for hypertension (140/90 mmHg) (Table 1).

**Table 1:** Baseline characteristic of participants who completed at least 6 weeks and anthropometric changes from baseline

	Rapeseed oil (W0 *n* = 24, W6 *n* = 24, W12 *n* = 21)		Sunflower oil (W0 *n* = 27, W6 *n* = 27, W12 *n* = 21)		Control (W0 *n* = 25, W6 *n* = 25, W12 *n* = 19)		*p* value ^1^
	Median (IQR)	Δ (Change)	Median (IQR)	Δ (Change)	Median (IQR)	Δ (Change)	
Weight, kg							
Week 0	86.0 (79.9–102.2)	–	83.4 (77.2–99.0)	–	89.6 (72.6–96.9)	–	0.69
Week 6	86.6 (78.9–103.0)	0.5 (− 0.25to 1.35)	84.4 (75.8–99.4)	0.4 (− 0.5 to 1.15)	86.8 (74.3–95.0)	0 (− 1.0 to 0.7)	0.74
Week 12	85.8* (76.3–104.2)	1.2 (− 0.5 to 2)	86.0 (77.0–102.9)	0.9 (− 1.1 to 1.6)	88.4 (75.2–94.6)	− 0.1 (− 1.6 to 0.4)	0.83
BMI, kg/m^2^							
Week 0	28.7 (27.3–31.0)	–	28.7 (26.3–31.9)	–	28.8 (26.4–31.1)	–	0.68
Week 6	29.4 (27.6–32.1)	0.2 (0.1 to 1.0)	29.0 (26.1–31.8)	0.1 (− 0.1 to 0.7)	29.7 (26.4–31.7)	0.2 (− 0.3 to 1.0)	0.84
Week 12	29.7 (27.6–32.0)	0.4 (0.1 to 1)	29.9 (27.1–32.5)	0.4 (− 0.1 to 1.1)	30.1 (25.7–31.7)	0.0 (− 0.7 to 0.9)	0.88
Waist Circumference, cm ♂/♀^2^							
Week 0	104 (102–111)/102 (95–105)	–	105 (94–115)/91 (87–105)	–	106 (104–108)/92 (85–100)	–	0.97/0.19
Week 6	103 (100–110)/95 (90–100)	− 2 (− 2 to − 1)/− 6 (− 8 to − 3)	101 (94–108)/87 (85–100)	− 1 (− 3 to 0)/− 2 (− 5 to 0)	104 (97–105)/87 (81–100)	− 3 (− 6 to − 1)/− 3 (− 4 to − 2)	0.81/0.26
Week 12	102 (98–106)/97 (91–103)	− 2 (− 2 to − 2)/− 4 (− 8 to − 1)	103 (99–108)/89 (85–95)	− 3 (− 3 to − 2)/− 1 (− 3 to − 1)	103 (97–105)/89 (81–96)	− 4 (− 5 to − 3)/− 4 (− 4 to − 3)	0.82/0.17
Systolic pressure^3^, mm Hg							
Week 0	131 (120–143)	–	126 (121–146)	–	134 (124–144)		0.60
Week 6	127 (120–138)	− 4.0 (− 8.5 to − 1.5)	128 (122–142)	1.0 (− 4.6 to 5.9)	130 (123–137)	− 5.0 (− 11.0 to 8.5)	0.93
Week 12	125 (120–142)	− 2.0 (− 7.5 to 5.9)	133 (125–143)	1.5 (− 6.8 to 11.8)	123** (116–135)	− 5.5 (− 10.8 to 0.1)	0.11
Diastolic pressure^3^, mm Hg							
Week 0	80 (75–92)	–	83 (79–89)	–	80 (76–89)	–	0.77
Week 6	81 (77–86)	− 3.0 (− 9.3 to 4.5)	84 (77–95)	− 1.5 (− 4.8 to 3.5)	79 (77–82)	− 0.5 (− 6.0 to 3.5)	0.39
Week 12	82 (76–87)	1.3 (4.8 to 9.1)	83 (77–87)	1.5 (− 3.5 to 5.0)	81 (75–85)	0.5 (− 3.8 to 4.8)	0.65

Data presented as medians (IQR)

^1^Kruskal-Wallis investigating concentration differences between groups

^2^Male/Female participants: Rapeseed (W0 *n* = 11/13, W6 *n* = 11/13, W12 *n* = 8/13), Sunflower (W0 *n* = 12/15, W6 *n* = 12/15, W12 *n* = 10/11), Control (W0 *n* = 11/14, W6 *n* = 11/14, W12 *n* = 9/10)

^3^Blood pressure data missing for 4 control, 3 RO and 2 SO participants at baseline, 4 control, 2 RO and 2 SO participants at week 6, and 2 RO and 1 SO participants at week 12

*Indicates a significant difference from baseline (*p* < 0.05)

**Indicates a significant difference from baseline and week 6 (*p* < 0.05)

### Dietary intake

Dietary intake did not vary between groups at baseline with no differences in energy intake, percentage energy from carbohydrates, protein, fat and saturated fat when assessed by food diaries (Table 2) or via FFQ (Table 3). Median saturated fat intake at baseline assessed by food diaries was 14%, 13% and 11% of energy for RO, SO and CON groups respectively.

**Table 2:** Two-day dietary intake (food diaries) assessed at baseline

	Rapeseed oil (*n* = 28)	Sunflower (*n* = 27)	Control (*n* = 27)	*p* value^1^
	Median (IQR)	Median (IQR)	Median (IQR)	
Energy (kcal)	1921 (1473–2380)	1795 (1358–2219)	1948 (1641–2285)	0.83
CHO (%E)	46 (42–54)	44 (38–50)	43 (39–48)	0.22
Protein (%E)	15 (13–20)	17 (15–20)	17 (16–19)	0.72
Fat (%E)	35 (30–39)	34 (30–43)	36 (33–40)	0.58
SFA (%E)	14 (11–15)	13 (11–17)	11 (10–15)	0.55
MUFA (%E)	12 (9–13)	10 (8–13)	12 (9–13)	0.40
MUFA (/g fat)	0.34 (0.29–0.37)	0.28 (0.24–0.33)	0.30 (0.26–0.36)	0.10
PUFA (%E)	4 (3–5)	4 (3–5)	5 (3–6)	0.82
PUFA (/g of fat)	0.12 (0.10–0.15)	0.11 (0.09–0.14)	0.13 (0.08–0.17)	0.54
Omega 3 (g)	0.6 (0.3–0.8)	0.6 (0.3–0.9)	0.8 (0.5–1.2)	0.20
Omega 6 (g)	3.6 (2.7–6.5)	2.5 (1.5–4.6)	4.4 (3.0–5.7)	0.10

*CHO* carbohydrate, *SFA* saturated fat, *MUFA* mono-unsaturated fatty acids, *PUFA* polyunsaturated fatty acids, *%E* percentage of total energy intake

^1^Kruskal-Wallis investigating concentration differences between groups at baseline. 1, 2, 4 participants in RO, SO and control groups respectively did not complete baseline food diaries

**Table 3:** Habitual dietary intake and habitual dietary oil assessed using FFQ at baseline by intervention group

	Rapeseed oil (*n* = 27)		Sunflower oil (*n* = 26)		Control (*n* = 23)		*p* value^5^
	Median (IQR)		Median (IQR)		Median (IQR)		
Energy (kcal/d)	1490	(1351–2013)	1678	(1420–2288)	1776	(1344–2146)	0.51
Carbohydrate (%E)	45	(40–49)	45	(40–47)	43	(37–47)	0.52
Protein (%E)	22	(19–23)	21	(20–23)	23	(21–25)	0.44
Fat (%E)	34	(31–36)	34	(29–39)	37	(32–40)	0.53
SFA (%E)	13	(11–15)	12	(12–14)	13	(12–16)	0.69
MUFA (%E)	12	(11–14)	12	(10–14)	13	(11–15)	0.5
PUFA (%E)	6	(5–7)	6	(5–7)	6	(5–8)	0.6
Habitual oil type^1^	*n*	%	*n*	%	*n*	%	
Olive oil^2^	17	65	18	72	14	78	0.88
Rapeseed oil	1	4	2	8	1	6	0.79
Other^3^	8	31	4	16	1	6	0.40
Habitual spread^4^	*n*	%	*n*	%	*n*	%	
Butter	10	37	14	54	8	35	0.33
Hard spreads	0	0	0	0	1	4	0.32
Soft spreads (polyunsaturated)	2	7	1	4	2	9	0.78
Low fat spreads	0	0	1	4	1	4	0.57
Very low-fat spreads	1	4	3	12	0	0	0.18
No habitual spread	14	52	7	27	11	52	0.25

*CHO* carbohydrate, *SFA* saturated fat, *MUFA* mono-unsaturated fatty acids, *PUFA* polyunsaturated fatty acids, *%E* percentage of total energy intake

^1^Defined in FFQ as cooking oil used “most of the time”

^2^High MUFA Oil: Virgin/Extra virgin olive oil or olive oil

^3^Sunflower oil, Coconut oil, Groundnut oil, other vegetable oil

^4^Defined as consumed at least 5–6/week, participants could select more than one option; assessed using Chi-squared

^5^Mann–Whitney *U* for differences in medians and fisher’s exact test investigating differences in proportions between groups at baseline

At baseline, the most consumed spread was butter with 38%, 52% and 28% of RO, SO and CON groups indicating they consume butter at least five times per week. There were no differences between groups in the consumption of butter, hard spreads, soft spread containing polyunsaturated fats, low or very low-fat spreads. The most habitually used cooking oil was olive oil (RO 65%, SO 72%, CON 78%). The second most habitually consumed oil was RO with 4%, 8% and 6% of RO, SO and CON groups respectively. Since oil incorporation in the diet was not prescriptive in term of which item to replace, it is only possible to broadly estimate the change in fatty acid profiles of the diet potentially achieved by the substitution. Based on baseline food diaries, assuming that total fat intake remained unchanged and a proportional replacement of fatty acid, the intervention would lead to moderate impact on saturated fat intakes (− 18% for RO, − 17% for SO), MUFA (19% for RO, 0% for SO) and more substantial impact of PUFA (24% for RO, 108% for SO), with no change in the control group.

### Adherence

After 6 weeks, 94% (*n* = 48/51) of participants in RO and SO groups were consuming at least 18 mL of oil per day (RO: 92%, *n* = 22/24 and SO: 96%, *n* = 26/27) when assessed based on weight of oil returned. After 12-week, adherence was 81% (*n* = 34/44) (RO: 76%, *n* = 16/21 and SO: 86% *n* = 16/21).

### Proteomics biomarkers

**Fig. 2:**
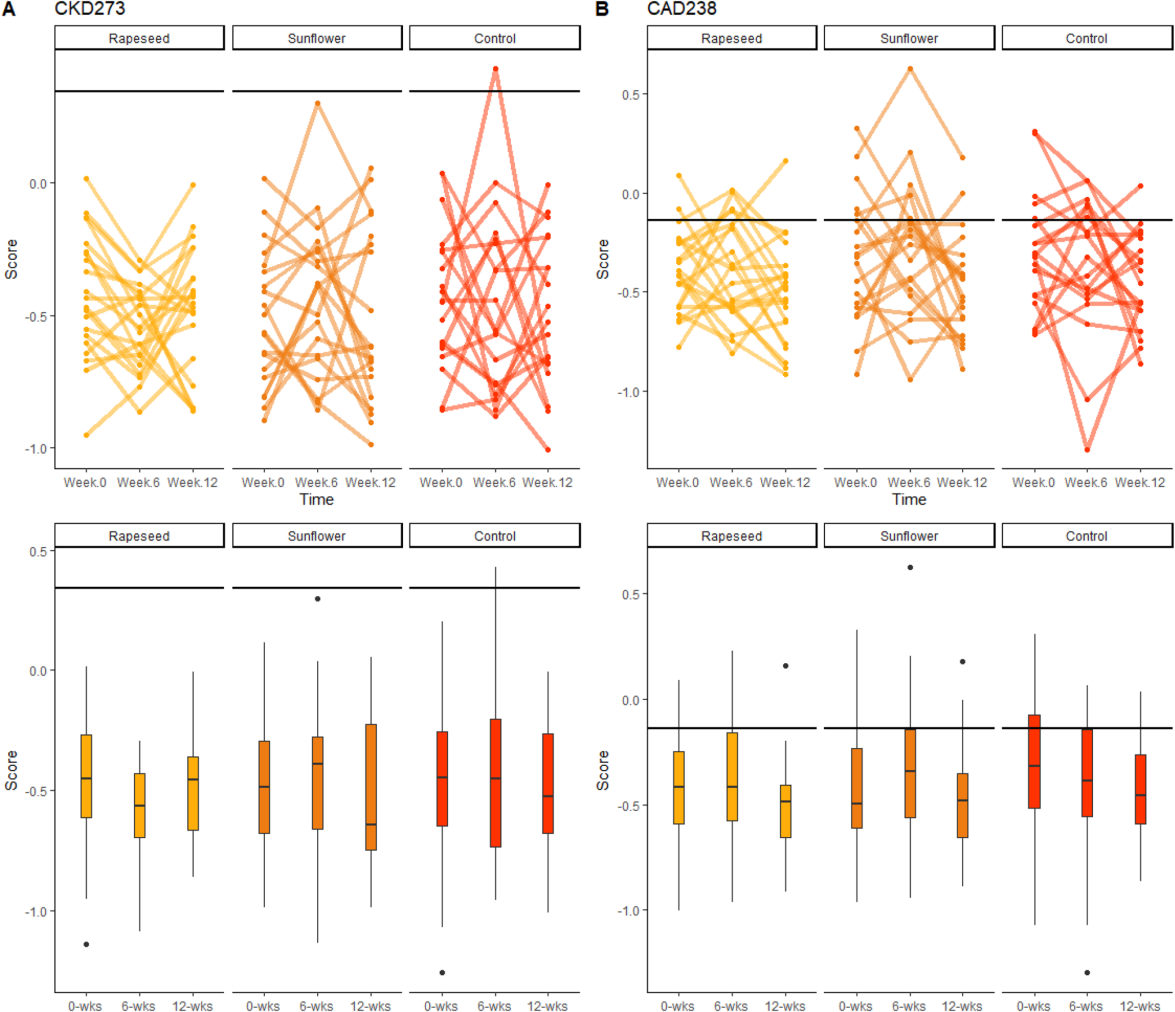
Urinary proteomic biomarker scores for coronary artery disease (CAD238), chronic kidney disease (CKD273) at weeks 0, 6 and 12 of the intervention. Data are presented as medians (IQR). Horizontal line indicates cut-off value for biomarker score (CKD273: 0.343, CAD238: 0.428). ^1^Kruskal-Wallis investigating concentration differences between groups at baseline. One-way ANCOVA investigated scoring differences between groups at weeks 6 and 12, after adjusting for baseline. ^2^Kruskal-Wallis investigating differences between groups in concentration change from baseline at weeks 6 and 12. **A** CKD273, **B** CAD238

The proteomic biomarkers were assessed in all participants who completed at least 6 weeks of the intervention. There were no differences in change between groups between time points or at any given timepoint for proteomic CAD or CKD (Fig. 2, Table 4). No detectable within-group differences were observed in CAD or CKD scores for RO, SO, or CON.

**Table 4:** Urinary proteomic biomarker scores for coronary artery disease (CAD238) and chronic kidney disease (CKD273) at week 0, 6 and 12 of the intervention

	Rapeseed oil (*n* = 21)		Sunflower oil (*n* = 21)		Control (*n* = 19)		*p* score^1^	*p* change^2^
	Score	Δ (change)	Score	Δ (change)	Score	Δ (change)		
CAD238								
Baseline	− 0.4 ± 0.2	–	− 0.4 ± 0.3	–	− 0.3 ± 0.3	–	0.37	–
Week 6	− 0.4 ± 0.2	0.0 ± 0.3	− 0.3 ± 0.3	0.0 ± 0.3	− 0.4 ± 0.3	− 0.1 ± 0.3	0.17	0.07
Week 12	− 0.5 ± 0.2	− 0.1 ± 0.2	− 0.4 ± 0.2	− 0.1 ± 0.3	− 0.4 ± 0.2	− 0.1 ± 0.3	0.63	0.95
CKD273								
Baseline	− 0.4 ± 0.2	–	− 0.5 ± 0.2	–	− 0.4 ± 0.3	–	0.96	–
Week 6	− 0. 5 ± 0.1	− 0.1 ± 0.3	− 0.5 ± 0.3	0.0 ± 0.2	− 0.4 ± 0.3	0.0 ± 0.3	0.06	0.10
Week 12	− 0.4 ± 0.2	0.0 ± 0.3	− 0.5 ± 0.3	0.0 ± 0.3	− 0.4 ± 0.2	0.0 ± 0.3	0.99	0.79

Data are presented as mean ± SD

^1^One-way ANOVA investigating scoring differences between groups at baseline. One-way ANCOVA investigated scoring differences between groups at weeks 6 and 12, after adjusting for baseline

^2^One-way ANOVA was used to investigate differences between groups in mean scoring change from baseline at weeks 6 and 12

*Indicates a significant difference from baseline (*p* < 0.05; none observed in this table)

The proportion of participants above the cut-off value for CKD did not change over time for any of the groups. At baseline, no participants were above the cut-off score for CKD (0.343). All participants in the RO and CON groups remained below the cut-off for the duration of the intervention, however, one participant in the CON group had a CKD score of 0.428 after 6 weeks. All participants were below the cut-off score at 12 weeks.

At baseline, 16 participants (21%) were above the cut-off of -0.140 for CAD, 13% (*n* = 3) in the RO group, 19% (*n* = 5) in the SO group, 31% (*n* = 8), in the CON group. The proportion of participants above the CAD cut-off score did not change significantly over time in any of the groups: After 6-weeks, *n* = 7 participants in the RO group, *n* = 6 in the SO group and *n* = 7 in the CON group were above the cut-off for CAD. After 12 weeks *n* = 4 participants were above − 0.140 score for CAD (1 RO, 2 SO and 1 CON).

### Changes in anthropometry over the intervention period

Anthropometric measures were assessed in all participants; however blood pressure data is missing for 9 completers at baseline (3 RO, 2 SO, 4 CON), 8 at week 6 (2 RO, 2 SO, 4 CON) and 3 (2 RO, 1 CON) at week 12. There was no difference observed in body weight, BMI, systolic or diastolic blood pressure between groups after 6 or 12 weeks of the intervention (Table 1). After 12 weeks a small decrease in body weight was observed in the RO group, from 86.0 kg (79.9–102.2) at week 0 to 85.6 kg (76.3–104.2) at week 12  (*p* = 0.032). A decrease in median systolic blood pressure was observed in the CON group after 12 weeks from 134 mmHg (124–144) to 123 mmHg (116–135) (*p* = 0.046). No other changes were observed within groups at 6 or 12 weeks.

### Plasma biomarkers

**Fig. 3:**
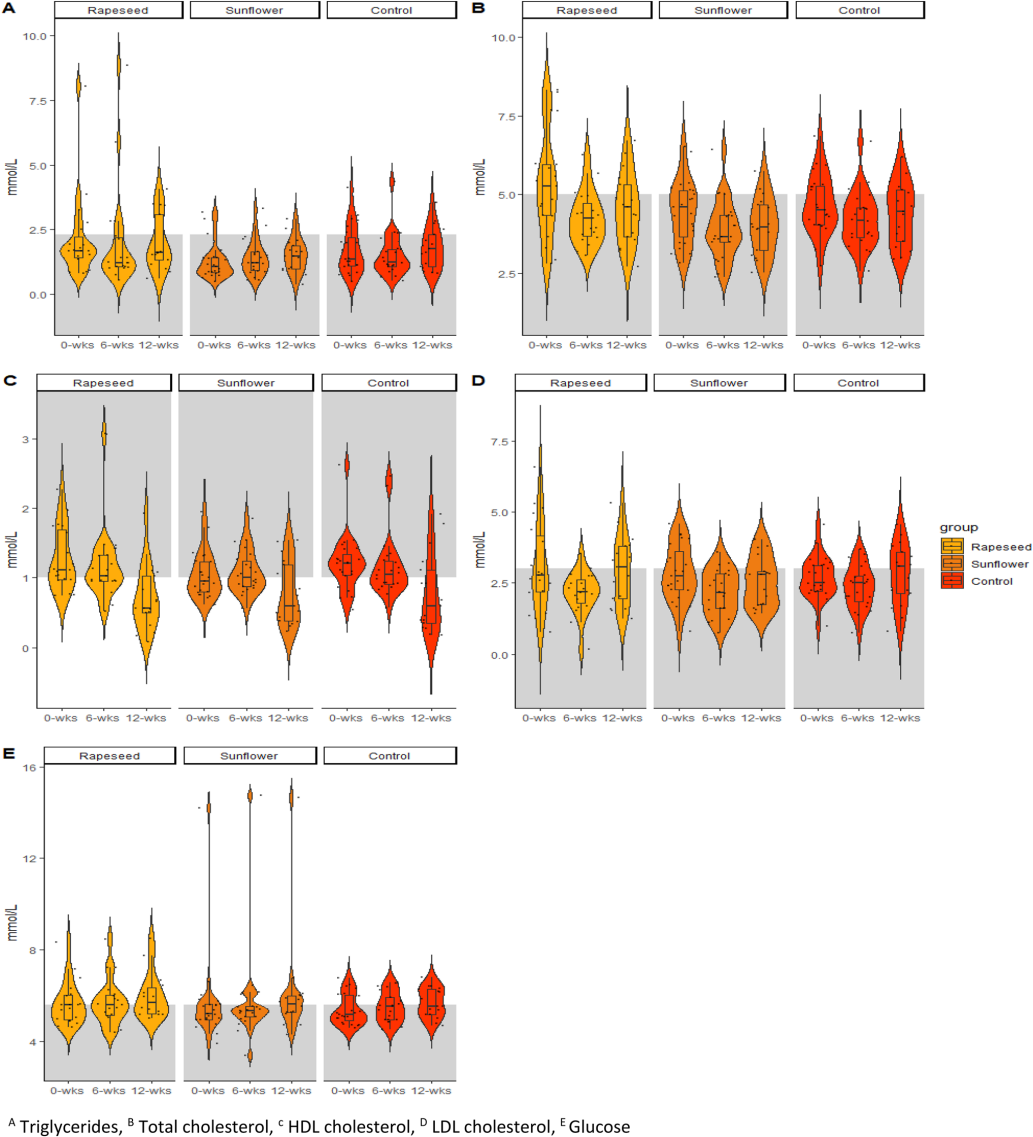
Plasma lipid concentrations (triglycerides, total cholesterol, HDL cholesterol, LDL cholesterol and fasting blood glucose) at week 0, 6 and 12 of the intervention. Grey area indicates normal range for each biomarker. **A** Triglycerides, **B** total cholesterol, **C** HDL cholesterol, **D** LDL cholesterol, **E** glucose. Plasma samples were missing from 10 participants at baseline (4 RO, 3 SO, 3 control), 14 participants at week 6 (5 RO, 5 SO, 4 control) and 10 participants at week 12 (4 RO, 4 SO, 2 control)

Plasma samples were missing from 8 completers at baseline (3 RO, 3 SO, 2 CON), 11 participants at week 6 (5 RO, 3 SO, 3 CON) and 9 participants at week 12 (4 RO, 3 SO, 2 CON), consequently data for these participants is missing from the analysis. Baseline plasma TC, HDL-C, LDL-C or glucose concentrations did not differ between groups. However, plasma TG concentration was higher in the RO group (1.7 mmol/L, IQR 1.4–2.2) compared to the SO group (1.1 mmol/L, IQR 0.8–1.4) (*p* = 0.03) (Fig. 3, Supplementary Table 1). No detectable differences in change in TC, TG, blood glucose, HDL-C or LDL-C were observed between groups at 6 or 12 weeks of the intervention.

Small within-group changes were noted: TC decreased in the RO group from 5.3 mmol/L (IQR 4.3–6.0) to 4.3 mmol/L (IQR 3.7–4.7) at 6 weeks and in the SO group from 4.6 mmol/L (IQR 3.7–5.1) to 3.7 mmol/L (IQR 3.5–4.7). These differences did not persist after 12 weeks.

There was no within-group change for HDL cholesterol levels after 6 weeks for any group; however, a decrease was noted from 1.1 mmol/L (IQR 1.0–1.7) to 0.6 mmol/L (IQR 0.5–1.0) in RO and 1.0 mmol/L (IQR 0.8–1.2) to 0.6 mmol/L (IQR 0.4–1.2) in SO group, after 12 weeks. Meanwhile, blood glucose increased in the CON group at 12 weeks (5.2 mmol/L, IQR 4.9–6.0 to 5.5 mmol/L (IQR 5.2–6.3). No other within-group differences in glucose concentration were observed.

### Oxidative stress and inflammatory biomarkers

There were no changes between groups or difference within group over time for fructosamine, AGEs or sRAGE levels (Table 5). After 12 weeks, median 8-isoprostane concentration was higher in SO group (1489 pg/mg IQR 1108–1902) compared to RO (972 pg/mg IQR 731–1378) and CON (888 pg/mg IQR 755–1072) (*p* = 0.03, Table 5), without difference between group in the change from baseline.

**Table 5:** Plasma concentrations of oxidative stress biomarkers (fructosamine, AGE, sRAGE and 8-isoprostanes) at weeks 0, 6, 12 of the intervention

	Rapeseed oil		Sunflower oil		Control		*P*meas^1^	*P*change^2^
	Concentration	ΔChange	Concentration	ΔChange	Concentration	ΔChange		
Fructosamine (mM)								
Baseline	0.5 (0.4–0.6)	–	0.4 (0.4–0.5)	–	0.4 (0.3–0.5)	–	0.26	–
Week 6	0.5 (0.4–0.5)	0.0 (− 0.1 to 0.0)	0.4 (0.3–0.5)	0.0 (− 0.1 to 0.0)	0.4 (0.4–0.6)	0.0 (0.0 to 0.1)	0.18	0.20
Week 12	0.5 (0.4–0.5)	0.0 (− 0.1 to 0.1)	0.4 (0.4–0.5)	0.0 (− 0.1 to 0.1)	0.5 (0.4–0.5)	0.0 (0.0 to 0.1)	0.45	0.72
AGE (AU)								
Baseline	202.8 (164.7–286.9)	–	181.0 (143.5–215.2)	–	201.6 (159.9–239.6)	–	0.46	–
Week 6	191.9 (170.4–248.1)	− 11.6 (− 50.7 to 25.9)	192.1 (159.8–227.8)	7.5 (− 21.5 to 28.5)	198.2 (172.7–221.9)	1.5 (− 36.3 to 43.1)	0.80	0.66
Week 12	245.0 (210.8–315.7)	12.8 (− 32.5 to 117.5)	200.7 (147.9–214.9)	− 2.7 (− 36.3 to 19.7)	214.1 (181.3–279.6)	− 1 (− 38.9 to 40.3)	0.17	0.60
sRAGE (ng/ml)								
Baseline	34.5 (28–39.4)	–	43.0 (30.3–56.6)	–	36.0 (27.5–46.7)	–	0.30	-
Week 6	28.9 (20.8–38.5)	− 2.4 (− 10.2 to 0.5)	36.9 (30.8–46.8)	− 0.4 (− 26.4 to 12.5)	30.2 (25.9–42.5)	− 0.4 (− 7.9 to 3.3)	0.13	0.71
Week 12	25.9 (20.5–34.8)	− 0.9 (− 9.7 to 2.9)	28.1 (25.6–50.2)	− 1.2 (− 19.3 to 6.3)	26.4 (22.9–36.7)	− 2.4 (− 15.2 to 0.8)	0.66	0.93
8-Isoprostanes (pg/mg creatinine)								
Baseline	1101.5 (640.4–1416.4)	–	1239.3 (1023.1–1613.7)	–	1027.1 (776.2–1341.7)	–	0.27	–
Week 6	1063.1 (811.7–1298.4)	− 138.9 (− 272.8 to 289.0)	978.31 (637.3–1415.7)	− 235.6 (− 557 to 114.6)	958.1 (773.3–1334.1)	23.3 (− 201.9 to 250.7)	0.97	0.11
Week 12	971.8 (731.3–1378.1)	60.1 (− 271.8 to 373.6)	1488.6 (1108.3–1902.0)	− 84.0 (− 311.3 to 253.2)	888.0 (754.8–1071.8)	31.8 (− 289.2 to 220.7)	**0.03**	0.68

Data are presented as medians (IQR)

^1^*P*meas: Kruskal–Wallis tested differences between groups at baseline. One-way ANCOVA tested differences between groups at weeks 6 and 12, after adjusting for baseline values

^2^*P*change: One-way ANOVA tested differences between groups in concentration changes from baseline at weeks 6 and 12. Friedman’s two-way analysis of variance was used to assess differences within groups. Plasma samples were missing from 10 participants at baseline (4 RO, 3 SO, 3 control), 14 participants at week 6 (5 RO, 5 SO, 4 control) and 10 participants at week 12 (4 RO, 4 SO, 2 control). *Indicates a significant difference from baseline, within group (*p* < 0.05; none in this table)

At baseline, no participants were found to have elevated concentrations of IL-6 and TNF-α. Values remained unchanged at weeks 6 and 12, with no difference between groups. No within-group changes or between-group differences in MMP-9 concentration were observed (Table 6).

**Table 6:** Plasma concentrations of inflammatory biomarkers (TNF-α, IL-6 and MMP-9) at weeks 0, 6 and 12 of the intervention

	Rapeseed oil (*n* = 21)		Sunflower oil (*n* = 21)		Control (*n* = 19)		*p* score^1^	*p* change^2^
	Score	Δ (change)	Score	Δ (change)	Score	Δ (change)		
**IL-6 (pg/mL)**								
Baseline	1.2	–	0.2	–	1.2	–	0.38	–
Week 6	1.2	0.0	1.2	0.0	1.2	0.0	0.56	0.37
Week 12	1.2	0.0	1.2	0.0	1.2	0.0	0.34	0.23
**TNF-α (pg/mL)**								
Baseline	1.8 (1.8–2.7)	–	1.8 (1.8–2.6)	–	2.0 (1.8–2.7)	–	0.59	–
Week 6	1.8 (1.8–2.1)	0.0	1.8 (1.8–3.4)	0.0 (0.0 to 0.5)	1.8 (1.8–2.7)	0.0 (− 0.2 to 0.0)	0.64	0.75
Week 12	1.8 (1.8–2.1)	0.0	1.8	0.0 (− 0.3 to 0.0)	1.8 (1.8–2.7)	0.0 (− 0.2 to 0.0)	0.92	0.51
**MMP-9 (ng/ml)**								
Baseline	103.3 (81.8–122.5)	–	100.0 (77.1–134.0)	–	81.1 (67.1–123.2)	–	0.60	
Week 6	95.1 (79.2–123.2)	3.6 (− 11.4 to 23.9)	91.2 (72.3–113.0)	− 5.0 (− 28.3 to 14.5)	83.9 (57.2–116.3)	− 1.1 (− 23.1 to 34.9)	0.78	0.58
Week 12	74.4 (63.1–126.6)	− 8.3 (− 39.0 to 20.2)	87.7 (67.2–96.6)	− 27.6 (− 64.5 to 2.2)	75.0 (56.9–119.0)	− 9.7 (− 30.0 to 14.3)	0.99	0.31

Data are presented as medians (IQR)

^1^Kruskal–Wallis tested differences between groups at baseline. One-way ANCOVA investigated concentration differences between groups at weeks 6 and 12, after adjusting for baseline values. Statistical significance: *p*score < 0.05

^2^One-way ANOVA investigated differences between groups in concentration changes from baseline at weeks 6 and 12. Statistical significance: *p*change < 0.05. Friedman’s two-way analysis of variance was used to assess differences within groups. Plasma samples were missing from 10 participants at baseline (4 RO, 3 SO, 3 control), 14 participants at week 6 (5 RO, 5 SO, 4 control) and 10 participants at week 12 (4 RO, 4 SO, 2 control). *Indicated a significant difference from baseline (*p* < 0.05, none observed in this table)

## Discussion

This is the first randomised controlled trial of commonly consumed unsaturated fatty acid-rich oils using novel proteomic biomarkers of health as a measure of outcome. The use of urinary proteomic biomarkers allows the early (pre-symptomatic) study of changes in the actual development of a specific disease and is not just a risk factor for that disease. The use of these highly specific fingerprint-like biomarkers is a new tool in dietary intervention trials, where the aim is the prevention of damage caused by disease progression and improvement in markers of health. The utility of the urinary proteomics biomarkers in guiding intervention was recently demonstrated in a large prospective, randomized, multicentre clinical trial (PRIORITY) [31], where proteomic biomarkers for kidney disease (i.e. CKD273 classifier) were applied to stratify type 2 diabetes patients with normoalbuminuria into those predicted to progress towards chronic kidney disease and those with low risk for progression. The first group was randomised for treatment with spironolactone in comparison to placebo on top of standard therapy, with the goal to reduce the development of microalbuminuria. In the cohort comprised of 1775 participants, positive scoring of proteomics biomarkers was associated with a hazard ratio of 2.48 for development of microalbuminuria, and a hazard ratio of 3.5 for onset of CKD stage 3 [31]. However, treatment with spironolactone did not show benefits in preventing CKD progression [31].

In this study, use of urinary proteomic biomarkers did not indicate any difference between groups (RO, SO or CON), or change in CAD or CKD biomarkers after either 6 weeks or 12 weeks. This contrasts with our previous observations following supplemental intake of high or low polyphenol olive oil (20 g/day over 6 weeks), with both olive oils types lowering the CAD score over time within groups, and no difference between groups [13].

Our study sample size was defined to detect an effect (mean reduction in CAD score) as large as previously observed with olive oil [13], and as little as half of that effect, with a sample size of *n* = 100 allowing for a 15% drop-out. In this instance, recruitment did not achieve a sample size of *n* = 100, which means that the intervention design can only be used to test hypotheses related to an effect on proteomics biomarkers as large as the one observed with olive oil. In this instance, we did not detect the aforementioned larger effect.

Attrition at week 12 was large, almost double (27%) than that anticipated (drop-out rate at 6-week was 8%, within our initial projection). Attrition may have been due to difficulty to comply with the intervention for a long period. Some participants stated a dislike for the taste of uncooked oil. Inability to obtain blood samples from some participants contributed to the underpower of the study for secondary outcomes. However, our main outcome was based on urinary proteomic biomarkers, for which samples were obtained for all participants.

The population sample in the current study differed to that of the olive oil study [13], with participants generally older with a median age of 43 compared to ~ 30 years. In this study, BMI and waist circumference were selection criteria, whilst the olive oil study included healthy participants from the general public with no restrictions on weight. We hypothesised that individuals with higher BMI and waist circumference would have a proteomic biomarker score with greater scope for improvement. Our participants were self-reported healthy which corroborated with our baseline results showing CKD biomarkers lower than disease cut-off of 0.343 for CKD [29, 32]. Only 21% of all participants in our study had a baseline CAD score above the cut-off of − 0.140 for CAD [10]. Silva et al. [13] demonstrated that marked reductions can be observed even in healthy populations, and the lack of change in the CAD scores (ΔCAD: − 0.1 to 0.0) from baseline to weeks 6 and 12 cannot be attributed to our study population’s normal scores at baseline. Moreover, CAD238 is a sensitive biomarker for early detection of CVD with advanced ability to identify asymptomatic cases and predict disease risk, hence changes could have been detected in our study sample.

Dietary factors likely to modify study findings include habitual fat intake. At baseline, 71% of participants reported olive oil as their habitual cooking oil, with rapeseed oil as the second most consumed cooking oil, which could have blunted the effect of the replacement intervention. This observation is in disagreement with the literature demonstrating that olive oil is not the main habitual oil in the UK [15]. This can partly be explained by the fact that the sample was recruited based on age and body composition criteria, without further criterion related to other sociodemographic variables (income level and age group), that have been shown to influence olive oil intake in the UK [17].

Whilst our participants reported consuming MUFA-rich oils, the average MUFA consumption in our study group was 11.3%E, below the recommended intake of 13%E (12, 10 and 12%E in the RO, SO and CON groups respectively). Inversely, our study population had a habitual saturated fat intake above the recommended level of 11%E, at 12.6%E (14, 13 and 11%E in the RO, SO and CON groups respectively), in line with the average intake of the UK population (11.9%E in adults aged 19–64, 13.3% in adults aged over 65) [33]. The total fat consumption was in accordance with the recommendations at 35% (35%, 34% and 36% in the RO, SO and CON groups respectively).

Minor within-group effects were noted for blood lipids as proxy markers of cardiovascular diseases, but no between-group differences. After 6 weeks, a decrease in TC concentration was observed in both SO group (from 4.6 to 3.7 mmol/L) and RO group (from 5.3 to 4.3 mmol/L). This is in partial agreement with Salar et al. [34] who showed a reduction in serum TC (− 11.9 ± 9.2 mmol/L) with RO (30 g daily) but an increase in TC with SO (4.1 ± 19.2 mmol/L) after 8 weeks in postmenopausal participants with overweight, obesity and T2DM. While the contribution of the oils to phytosterol and polyphenolics intake was not defined in this study, a suggested MUFA-independent mechanism for the cholesterol-lowering effect of RO relates to its phytosterol content, with vegetable oils as sources of phytosterols in the diet [35]. Levels of phytosterol (contributed mostly to by sitosterol) are similar between RO (250 mg/100 g) and SO (290 mg/100 g), and higher than olive oil (114 mg/100 g) [36].

Our findings are in partial agreement with others who reported no difference in blood lipids from baseline after 6-month supplementation of MUFA-rich muffins [37]. Miller et al. attributed the lack of change in blood lipid concentrations to the normal lipid profiles of participants at baseline. Similarly, we followed a replacement study model and our cohort presented median plasma lipid concentrations within or close to optimal ranges at baseline.

The role of dietary fats in modulating oxidative stress and the risk of cardiometabolic diseases has been reviewed, with evidence that neither saturated nor MUFA-rich diets impact on isoprostane levels, while n-3 PUFA and trans-fatty acids may decrease and increase levels, respectively [38]. Plasma 8-isoprostane levels were higher at 12-week in the SO group (a source of *n*-6 PUFA) compared to the other two groups, an observation which was not mirrored when change from baseline was compared between groups. This observation may merit further investigation, replicating the study design with a broader range of oxidative stress markers, and objective assessment of red blood cell lipid composition.

The study design, a randomised controlled design with stratified randomisation, ensured the control of bias. Additionally, unlike other studies including an alternative dietary oil as a control group, our study included a group following their habitual diet with no changes to dietary oil consumption. This also meant that it was not possible to blind participants and researchers to allocation to the control group as no intervention was provided.

The inclusion of both sexes with a broad age group increased the generalisability of the study and its application for dietary guideline recommendations. However, it might have increased the inter-individual variability in the response to dietary fat intake as well as sex differences for CVD risk. Participants were requested to follow the same diet and maintain a dietary record 2 days preceding each visit, for normalisation. We used a replacement model as opposed to supplementation used in other studies [34]. A replacement model can address concerns regarding weight gain and adverse effects on health from supplementation with excess dietary oils in populations at higher risk of CVD. This was relevant in this sample of individuals with obesity and overweight, consequently there was no difference in weight change observed between the three groups. Our replacement strategy was non-prescriptive, which may have been a source of variability in the execution of the protocol by the participants. Additionally, we could not control for other oils consumed during the study period. Dietary oil intake is usually hidden, especially when oils are used for cooking [39].

The changes in CAD biomarkers observed in our previous study [13] were unmatched for either oil in this intervention. Further research is needed to understand which components of dietary oils (lipids and bioactives) have a protective effect on cardiometabolic health, with a view to further inform dietary guidelines.

## Supplementary Information

Below is the link to the electronic supplementary material.

Supplementary file1 (DOCX 20 kb)
