# Supplementary material for: No evidence of differential impact of sunflower and rapeseed oil on biomarkers of coronary artery disease or chronic kidney disease in healthy adults with overweight and obesity: result from a randomised control trial
Source: Eur J Nutr. 2022 Apr 5;61(6):3119–33. doi: 10.1007/s00394-022-02810-5 (PMC9363295; doi:10.1007/s00394-022-02810-5)
Supplement: Supplementary file 1 — Supplementary file1 (DOCX 20 kb) [file 394_2022_2810_MOESM1_ESM.docx]

Supplementary Table 1: Plasma lipid concentrations (triglycerides, total cholesterol, HDL cholesterol, LDL cholesterol and glucose) at week 0, 6 and 12 of the intervention

|  | **Rapeseed Oil** | **Sunflower Oil** | **Control** | **P-value** |
| --- | --- | --- | --- | --- |
| **Triglycerides (mmol/L)** | | | |  |
| Baseline | 1.7 (1.4-2.2) | 1.1 (0.8-1.4) | 1.4 (1.1-2.2) | **0.03** |
| Week 6 | 1.2 (1.1-2.2) | 1.2 (0.9-1.6) | 1.2 (1.1-1.7) | 0.85 |
| Week 12 | 1.6 (1.3-3.1) | 1.5 (1.0-1.9) | 1.8 (1.1-2.3) | 0.51 |
| **Total cholesterol (mmol/L)** | | | |  |
| Baseline | 5.3 (4.3-6.0) | 4.6 (3.7-5.1) | 4.5 (4.0-5.3) | 0.17 |
| Week 6 | **4.3 (3.7-4.7)*** | **3.7 (3.5-4.3)*** | 4.2 (3.6-4.6) | 0.30 |
| Week 12 | 4.6 (3.7-5.3) | 4.0 (3.2-4.7) | 4.5 (3.5-5.2) | 0.35 |
| **HDL-C (mmol/L)** | | | |  |
| Baseline | 1.1 (1.0-1.7) | 1.0 (0.8-1.2) | 1.2 (1.0-1.3) | 0.12 |
| Week 6 | 1.0 (1.0-1.3) | 1.0 (0.9-1.2) | 1.1 (0.9-1.2) | 0.81 |
| Week 12 | **0.6 (0.5-1.0)*** | **0.6 (0.4-1.2)*** | 0.6 (0.4-1.1) | 0.91 |
| **LDL-C (mmol/L)** | | | |  |
| Baseline | 2.8 (2.2-4.2) | 2.7 (2.3-3.6) | 2.5 (2.2-3.1) | 0.82 |
| Week 6 | 2.2 (1.8-2.6) | 2.2 (1.6-2.8) | 2.5 (1.8-2.8) | 0.76 |
| Week 12 | 3.1 (2.0-3.8) | 2.8 (1.8-2.9) | 3.1 (2.1-3.6) | 0.66 |
| **Glucose (mmol/L)** | | | |  |
| Baseline | 5.6 (4.9-6.0) | 5.2 (4.9-5.6) | 5.2 (4.9-6.0) | 0.63 |
| Week 6 | 5.6 (5.1-6.0) | 5.3 (5.1-5.5) | 5.5 (4.9-5.9) | 0.61 |
| Week 12 | 5.7 (5.2-6.4) | 5.6 (5.3-6.0) | **5.5 (5.2-6.3)*** | 0.76 |

Data are presented as medians (IQR). Kruskal-Wallis for non-parametric variables investigating scoring differences between groups. Friedman’s two-way analysis of variance was used to assess differences within groups. The significance level is 0.05. *Indicated a significant difference from baseline. Plasma samples were missing from 10 participants at baseline (4 RO, 3 SO, 3 control), 14 participants at week 6 (5 RO, 5 SO, 4 control) and 10 participants at week 12 (4 RO, 4 SO, 2 control).
